# Blood concentration of bupivacaine and duration of sensory and motor block following ultrasound-guided femoral and sciatic nerve blocks in dogs

**DOI:** 10.1371/journal.pone.0193400

**Published:** 2018-03-05

**Authors:** Michéal O. Cathasaigh, Matt R. Read, Aylin Atilla, Teresa Schiller, Grace P. S. Kwong

**Affiliations:** Faculty of Veterinary Medicine, University of Calgary, Calgary, Canada; University of Bari, ITALY

## Abstract

Peripheral nerve blocks are becoming increasingly popular for perioperative use as anesthetics and analgesics in small animals. This prospective study was performed to investigate the duration of motor and sensory blockade following use of bupivacaine for ultrasound-guided femoral and sciatic nerve blocks in dogs and to measure the plasma concentrations of bupivacaine that result from these procedures. Six dogs were anesthetized twice using a randomized cross-over design. At the first anesthetic, dogs were assigned to receive either an ultrasound-guided femoral nerve block or sciatic nerve block with 0.15 mL kg^-1^ of bupivacaine 0.5%. Two months later, the other nerve block was performed during a second anesthetic. At 5, 10, 15, 20, 30 and 60 minutes after injection, arterial blood samples were collected for laboratory measurement of bupivacaine. After 60 minutes, dogs were recovered from anesthesia. Starting at two hours post-injection, video-recordings of the dogs were made every two hours for 24 hours. The videos were randomized and the degree of motor and sensory blockade was evaluated using a three-point scoring system (0 = no effect, 1 = mild effect, 2 = complete blockade) by two blinded assessors. The median (range) times to full recovery from motor blockade were 11 (6–14) hours (femoral) and 12 (4–18) hours (sciatic), and 15 (10–18) hours (femoral) and 10 (4–12) hours (sciatic) for sensory blockade. There were no differences in the median times to functional recovery for the two techniques. Plasma concentrations of bupivacaine were no different following the blocks and were less than 0.78 μg mL^-1^ at all times. These results suggest that these ultrasound-guided nerve blocks do not result in potentially toxic systemic levels of local anesthetic and that their duration of action is useful for providing anesthesia and analgesia for pelvic limb procedures.

## Introduction

Peripheral nerve blocks (PNBs) involve the injection of local anesthetic drugs around nerves outside of the vertebral column in order to induce muscle relaxation and/or prevent pain transmission. Although it has long been appreciated that using perioperative regional anesthesia instead of, or in combination with, general anesthesia has numerous benefits in people, the potential benefits of using PNBs over other techniques such as systemically-administered opioids or epidural anesthesia have only recently been investigated in small animals [1–4].

Certain small animal nerve block techniques use straight forward surface anatomical landmarks as reference points for presumed nerve location and, as a result, are typically performed using a ‘blind’ approach. In the situation where nerves of the thoracic or pelvic limb are to be blocked, the peripheral nerves of interest are less likely to be superficially located and they are frequently found in close proximity to other important structures such as blood vessels, the abdomen, or the vertebral canal. Use of more advanced techniques such as peripheral nerve electrostimulation and ultrasound have been evaluated for their abilities to enable and improve success rates of nerve blocks that can be used for a growing number of invasive and painful surgical procedures [5–17].

Use of ultrasound for performing peripheral nerve blocks has been investigated and used in people for over 20 years and is becoming increasingly popular for facilitating various regional anesthetic techniques and peripheral nerve blocks in small animals [5–12,14–17]. Ultrasound offers several advantages over blind and nerve stimulator-guided approaches, including the ability to visualize the procedure in real time while identifying the target nerve and manipulating the needle relative to the nerve and other important anatomical structures [5–8,15–17]. Since ultrasound also allows for real-time visualization of the spread of local anesthetic around the nerve, the anesthetic solution may be deposited more precisely, making it possible to use lower doses of local anesthetics than are required for the other techniques without negatively affecting the efficacy of the nerve block. As a result, these techniques may improve block success, reduce the need for multiple needle passes, reduce the risk of vascular puncture, and minimize performance time [5,7,8,15–17].

In dogs, pelvic limb innervation can include branches of the femoral nerve (FN), saphenous nerve (SaN), obturator nerve (ON), lateral cutaneous femoral nerve (LCFN) and sciatic nerve (ScN) [10,15,17–19]. Initial studies reported the anatomical considerations for blocking the two main nerves, the FN and the ScN, at different locations along their courses while subsequent studies described the volume of dye that is required to stain a desired length of target nerve in cadaver specimens and the technical aspects of using ultrasound and/or nerve stimulation to perform these peripheral nerve blocks [5,6,10,11]. Clinical studies have documented the effects of different FN and ScN blocks on intraoperative analgesia, recovery quality, and the incidence of side effects and complications related to their use for surgical procedures of the pelvic limb [1,2,3,4,9,17,20,21]. Based on these clinical studies in dogs, advantages of using PNBs over neuraxial or opioid-based methods of perioperative pain control include decreased inhaled anesthetic requirements, better maintenance of blood pressure during anesthesia, smoother recoveries from anesthesia, less post-operative urinary retention, and equivalent or superior postoperative analgesia.

The success of any peripheral nerve block depends on the accurate injection of local anesthetic around the nerves that serve the planned surgical site. Ultrasound-guided blockade of the ScN from a caudal approach at the mid-femur has been well described in dogs and is associated with high success rates [1,4–7]. Although initial investigations reported the results of blocking the SaN/FN at a location in the inguinal region of the pelvic limb, since the FN and ON are contiguous proximally within the iliopsoas muscle, some investigators have explored blocking these nerves at this more proximal location [10–17]. Interest in this alternative site is based on observations that, although the medial articular nerve arises from the SaN/ FN in the majority of dogs, in a small proportion of dogs, sensory innervation of the medial aspect of the limb may also receive contributions from the ON [18]. In dogs with that particular innervation pattern, injecting a local anesthetic solely around the SaN/ FN after it has already exited the muscular lacuna would not be expected to result in complete analgesia of the limb being operated on. Investigations have shown that injecting a local anesthetic around the lumbar plexus will result in two-in-one blockade of both the FN and the ON with a single injection, accounting for each of the potential sensory innervation patterns of the pelvic limb [10,13,15,17].

While the use of ultrasound and nerve stimulation for performing peripheral nerve blocks has been a growing area of research, there are still questions that need to be answered before widespread adoption of these techniques in small animal practice is realized. To date there is only a single report of the expected duration of action of bupivacaine following injection around the FN and ON within the iliopsoas muscle [16], and little is known about how quickly a local anesthetic might be absorbed from an intramuscular location (e.g. the iliopsoas muscle) compared to an interfascial site of administration (e.g. the ultrasound-guided caudal approach to the ScN).

This study was performed to answer these questions. We hypothesized that, following administration of the same dose of bupivacaine, injection into the iliopsoas muscle for the FN block will result in higher plasma concentrations of bupivacaine than injection of bupivacaine between the different fascial planes that surround the ScN. Further, we hypothesized that the duration of motor and sensory dysfunction would be shorter following FN block than ScN block as a result of higher relative local blood flow and more rapid uptake of the local anesthetic from the site of administration.

## Materials and methods

### Anesthesia

This study was approved by the University of Calgary Veterinary Sciences Animal Care Committee (#AC14-0158). Six Beagles (three male and three female; 4–7 yr; 8.8–11.5 kg) from the University of Calgary Teaching Herd were used in this study. Dogs were housed and maintained in accordance with Canadian Council on Animal Care guidelines and were returned to the Teaching Herd at the conclusion of the study. All dogs were considered to be healthy based on the results of physical examination, complete blood count and serum biochemistry. None of the animals had a history of any pelvic limb, neurological or musculoskeletal disorders. Each dog was anesthetized two times with an eight-week washout period between the two anesthetics. At the time of the first anesthetic, dogs were randomly assigned to receive either an ultrasound-guided femoral nerve block or an ultrasound-guided sciatic nerve block. The other peripheral nerve block was performed at the second anesthetic.

Prior to anesthesia, dogs were fasted for 12 hours but had free access to water. On the day of the study, dogs were delivered to the laboratory and each dog was weighed and assessed for any abnormalities not previously recognized. The hair over a cephalic vein was clipped and aseptically prepared and a 20-gauge intravenous catheter (BD Insyte, Becton Dickinson Infusion Therapy Systems Inc., Sandy, UT) was placed in the vein. Crystalloid fluids (Plasma-Lyte 148 Injection Baxter Corp., Mississauga, ON) were administered through the catheter at a rate of 10 mL kg^-1^ hour^-1^ by a fluid pump. Following intravenous catheterization, each dog was induced using an inhalant anesthetic technique with isoflurane (Isoflurane USP AErrane, Baxter Canada, Mississauga, ON) in oxygen that was delivered using a Bain (non-rebreathing) system (Small Animal Anesthetic Machine, Moduflex Optimax, Dispomed, Joliette, QC) and a tight-fitting mask over the dog’s muzzle. The flow of oxygen was maintained between 100–150 mL kg^-1^ minute^-1^ to prevent rebreathing. Once the dog was assessed to be at an appropriate depth to permit orotracheal intubation, the mask was removed and the dog was intubated and connected to the breathing circuit to maintain anesthesia. No other sedative or anesthetic agents were administered.

Following intubation, dogs were positioned in left lateral recumbency and patient monitors were connected. Patient monitoring (Lifewindow 6000V, Digicare Biomedical Technology Inc., Boynton Beach, FL) included continuous lead II electrocardiography, esophageal temperature, arterial oxygen saturation of hemoglobin, non-invasive oscillometric systemic blood pressure, and sidestream end-tidal carbon dioxide and inspired and expired anesthetic agent analysis. A 20-gauge intravenous catheter (BD Insyte, Becton Dickinson Infusion Therapy Systems Inc., Sandy, UT) was placed in the left dorsal pedal artery, capped (BD PRN Adapter, Becton Dickinson Infusion Therapy Systems Inc., Sandy, UT) and flushed with saline (Sodium Chloride Injection USP, Hospira, Montreal, QC) to facilitate intermittent blood collection (see below). Instrumentation of each dog was achieved within 10 minutes of induction.

A dedicated anesthetist adjusted the level of isoflurane throughout anesthesia to maintain a light surgical plane of anesthesia. During anesthesia, the anesthetist used standard clinical interventions to maintain each patient’s parameters within acceptable ranges, including use of mechanical ventilation (Veterinary Anesthesia Ventilator Model 2000IE, Hallowell EMC, Pittsfield, MA) to maintain normocapnea and a warming device (HotDog™ Patient Warming System, Augustine Biomedical + Design, Eden Prairie, MN) to maintain normothermia. Arterial hypotension was treated by lowering the level of inhalant anesthesia and/or providing a 5 mL kg^-1^ bolus of crystalloid fluids over ten minutes, as appropriate.

### Peripheral nerve blocks

Femoral and sciatic nerve blocks were performed using a combination of ultrasound and eletrostimulation using previously described techniques [5,12]. Prior to each block, hair over each target site was clipped and the skin was aseptically prepared for injection (Stanhexidine, chlorhexidine gluconate solution, Omega Laboratories Ltd., Montreal, QC; Isopropyl alcohol 70%, Canadian Alcohol Company, Scarborough, ON). Ultrasonography was performed using an 8–15 MHz linear transducer connected to a portable ultrasound unit (Edge™ ultrasound system, SonoSite, Inc. Bothwell, WA, USA). Adjustments in depth and gain were made to obtain the optimal view of each target nerve. Electrostimulation was achieved using a portable stimulator (Stimuplex^®^ HNS12, B.Braun Medical Inc., Bethlehem, PA) and insulated needles (UniPlex NanoLine, Pajunk GmbH Medizintechnologie, Germany). For each block, 0.15 mL kg^-1^ bupivacaine 0.5% (Marcaine^®^ 0.50%, Bupivacaine hydrochloride injection USP, Hospira, Montreal, QC) was administered perineurally. Injections were carried out under direct echographic visualization.

#### Femoral nerve block (ventral suprainguinal approach)

The dog was positioned in dorsal recumbency with the limb to be blocked extended caudally. The medial thigh and ventrolateral abdomen were clipped and aseptically prepared and a coupling agent was applied to the skin. The iliopsoas muscle was scanned in transverse (short-axis) orientation with the ultrasound transducer oriented perpendicular to the midline and positioned slightly cranial to the inguinal nipple with the orientation marker positioned medially. Once the external iliac vessels and the FN were identified, a 22-gauge 50 mm insulated needle was connected to a peripheral nerve stimulator and introduced percutaneously in a lateromedial direction into the iliopsoas muscle. The peripheral nerve stimulator was set to deliver a current intensity of 0.5 mA with a stimulation frequency of 1 Hz and pulse duration of 0.1 ms. The needle was advanced using an in-plane technique and ultrasound was used to monitor the progression of the needle towards the FN. Identification of the FN was confirmed when there was sonographic evidence that the needle tip was in close proximity to the nerve and contractions of the quadriceps muscle were elicited. The nerve stimulator was turned off and, after confirming that blood could not be aspirated and that there was minimal resistance to injection, 0.15 mL kg^-1^ of bupivacaine was slowly injected through the needle. The spread of the local anesthetic was observed in real-time using ultrasound and the needle was manipulated in order to disperse the local anesthetic around the FN. Administration of the injectate was discontinued and the needle repositioned if there was any perceived increase in resistance during injection. Once the total volume of bupivacaine was injected, the needle was withdrawn and the ultrasound transducer was removed from the skin.

#### Sciatic nerve block

The dog was positioned in left lateral recumbency with the right pelvic limb positioned uppermost in a natural orientation. An area over the lateral and caudal mid-thigh was clipped and aseptically prepared and a coupling agent was applied to the skin. The limb was scanned in transverse (short-axis) orientation with the ultrasound transducer placed on the lateral aspect of the limb, perpendicular to the biceps femoris muscle and distal to the ischiatic tuberosity. Once the ScN was identified, a 22-gauge 50 mm insulated needle was connected to a peripheral nerve stimulator and introduced through the skin from a caudal direction through the semimembranosus and semitendinosus muscles. The peripheral nerve stimulator was set to deliver a current intensity of 0.5 mA with a stimulation frequency of 1 Hz and pulse duration of 0.1 ms. The needle was advanced using an in-plane technique and ultrasound was used to monitor the progression of the needle towards the ScN. Identification of the ScN was confirmed when there was sonographic evidence that the needle tip was in close proximity to the nerve and extension and/or flexion of the tarsus was elicited (indicating tibial nerve and/or common peroneal nerve stimulation, respectively). The nerve stimulator was turned off and, after confirming that blood could not be aspirated and that there was minimal resistance to injection, 0.15 mL kg^-1^ of bupivacaine was slowly injected through the needle. The spread of the local anesthetic was observed in real time using ultrasound and the needle was manipulated in an attempt to disperse the local anesthetic around the ScN. Administration of the injectate was discontinued and the needle repositioned if there was any perceived increase in resistance during injection. Once the total volume of bupivacaine was injected, the needle was withdrawn and the ultrasound transducer was removed from the skin.

### Bupivacaine analysis

Immediately after completing the bupivacaine injection, the time was noted. At 5, 10, 15, 20, 30 and 60 minutes following each nerve block, 10 mL of arterial blood was collected for bupivacaine analysis. In three dogs, 10 mL of blood was also collected prior to performing the nerve blocks in order to serve as blank (untreated) samples for calibration and quality control at the laboratory. At each time point, 3 mL of blood was first withdrawn from the arterial catheter, accounting for the presence of saline flush that would be present and thus preventing the blood sample that would ultimately be sent to the laboratory from being diluted. Next, 10 mL of blood was slowly withdrawn over 30 seconds and placed into heparinized blood collection tubes (BD Vacutainer^®^ Lithium Heparin 95 USP units (6.0 mL), Becton Dickinson, Franklin Lakes, NJ). After each blood sample was obtained, the arterial catheter was flushed with 2 mL of saline to prevent clotting until the next sample was collected. Blood tubes were centrifuged within 30 minutes and the plasma was removed and placed into screw-top vials (Cryovial^®^, Simport, Beloeil, QC). Plasma samples were frozen at -80°C until being sent to the laboratory for drug analysis. Plasma bupivacaine concentrations were measured using a validated high-pressure liquid chromatography assay. The limit of quantification (LOQ) was 0.05 μg mL^-1^ and the limit of detection was at 0.01 μg mL^-1^. Plasma concentrations are reported as micrograms of drug per milliliter of plasma. After the final blood sample was collected, venous and arterial catheters were removed and the delivery of isoflurane was discontinued. Dogs were allowed to breathe 100% oxygen through the breathing circuit until they were extubated. For recovery, the dogs were placed onto blankets in a small pen for observation and temperature, pulse and respiration were intermittently monitored until the dogs were awake and standing.

### Monitoring of sensory and motor blockade

In order to evaluate the intensity and duration of each block, dogs were assessed for sensory and motor function every two hours for 24 hours, starting two hours following bupivacaine administration. Since the dogs were anesthetized for 60 minutes following performance of each block, onset time of the blocks was not assessed. At each assessment, dogs were placed into lateral recumbency on an examination table before a video camera to allow the interaction to be recorded and randomized for later review by two blinded assessors. To assess the degree of sensory blockade, a pair of rat-tooth forceps was used to pinch the skin at 2–3 pre-defined locations within the corresponding cutaneous areas of each nerve. The ScN was tested by pinching the skin over the lateral aspect of the distal thigh, the lateral aspect of the tarsus, and the lateral aspect of the fifth digit. The SaN (sensory branch of the FN) was tested by pinching the skin over the medial aspect of the stifle and the medial aspect of the tibia. Next, the dog was placed on the floor for assessment of its ability to use and weight-bear on the anesthetized limb when standing, turning, and walking 15 metres toward and away from a video camera on a non-slip floor. At the conclusion of the study, each of the individual video recordings (specific dog following a specific block at a specific time point; sensory vs. motor) were assigned a unique identifier and recordings were randomized.

Two small animal veterinary surgeons were invited to serve as blinded reviewers of these videos and were trained to use a previously described instrument to score limb function [7,8]. For each block, the minimum score was 0 when the animal had normal function and the maximum score was 2 when the animal had complete blockade. Before they were provided with all of the videos collected during the study, nine motor and nine sensory videos were relabeled by the primary investigators and provided to the assessors. Of the nine videos, three showed dogs with “normal” limb function (i.e. score = 0), three showed an “intermediate” level of motor or sensory blockade (i.e. score = 1) and three showed “complete” motor or sensory blockade (i.e. score = 2). Each rater was asked to independently score each video using the instrument and inter-rater agreement was calculated and compared to the known “gold standard” score of limb function for each video. Evaluation of the scores from this activity was made and, based on the high level of inter-rater agreement, further training was not required and the assessors were provided with the complete set of videos to review.

Each assessor independently scored the degree of sensory or motor function in each video using the scoring instrument. Motor function was assessed using a three-point scale based on observations of leg position and proprioception and the ability of the dog to use the anesthetized limb when walking and standing: 0, normal weight bearing; 1, deficient weight bearing due to partial motor block; 2, inability to support the anesthetized limb due to complete motor block. Sensory function was assessed using a three-point scale based on observations of responses to pinching the skin (e.g. avoidance, vocalization, withdrawal of the limb): 0, normal response; 1, decreased response due to partial sensory block; 2, no response where the sensory block was complete. Clinically relevant blocks were defined as scores ≥ 1. For the purposes of data analysis, dogs were considered to have return of normal motor or sensory function at the first time point when one or both of the blinded assessors assigned a score of “0” to the video.

### Statistical analysis

After fitting a mixed effects model, peak plasma bupivacaine concentrations were compared between blocks at each time point and within each block over time. Post hoc analysis was performed using a Bonferroni correction for multiple comparisons. Weighted kappa values (95% CI) were calculated to assess the degree of inter-rater agreement of block effect scores (pairwise ordinal data). A Wilcoxon signed rank test was used to test for differences in the median durations of clinically relevant motor and sensory deficits between the two blocks. A Wilcoxon rank sum test was used to test for differences in the median durations of clinically relevant motor and sensory deficits for each of the blocks. Analyses were performed using R version 3.3.3; ‘nlme’ package version 3.1–131 was used to fit a mixed effects model and ‘lsmeans’ package version 2.26–3 was used for post hoc analysis; ‘irr’ version 0.84 and ‘psych’ version 1.6.4 packages were used for measuring agreements. Values of *P* < 0.05 were considered statistically significant for all tests.

## Results

All six dogs had normal motor and sensory function at baseline and induction of anesthesia with isoflurane was smooth and rapid in all cases. A total of 12 perineural blocks were performed and no complications (i.e. resistance to injection or blood aspiration) were observed during performance of the procedures. No other complications or adverse effects were encountered during the study and all dogs recovered uneventfully and did not show signs of neurological disorders as a result of a nerve injury.

**Fig 1:**
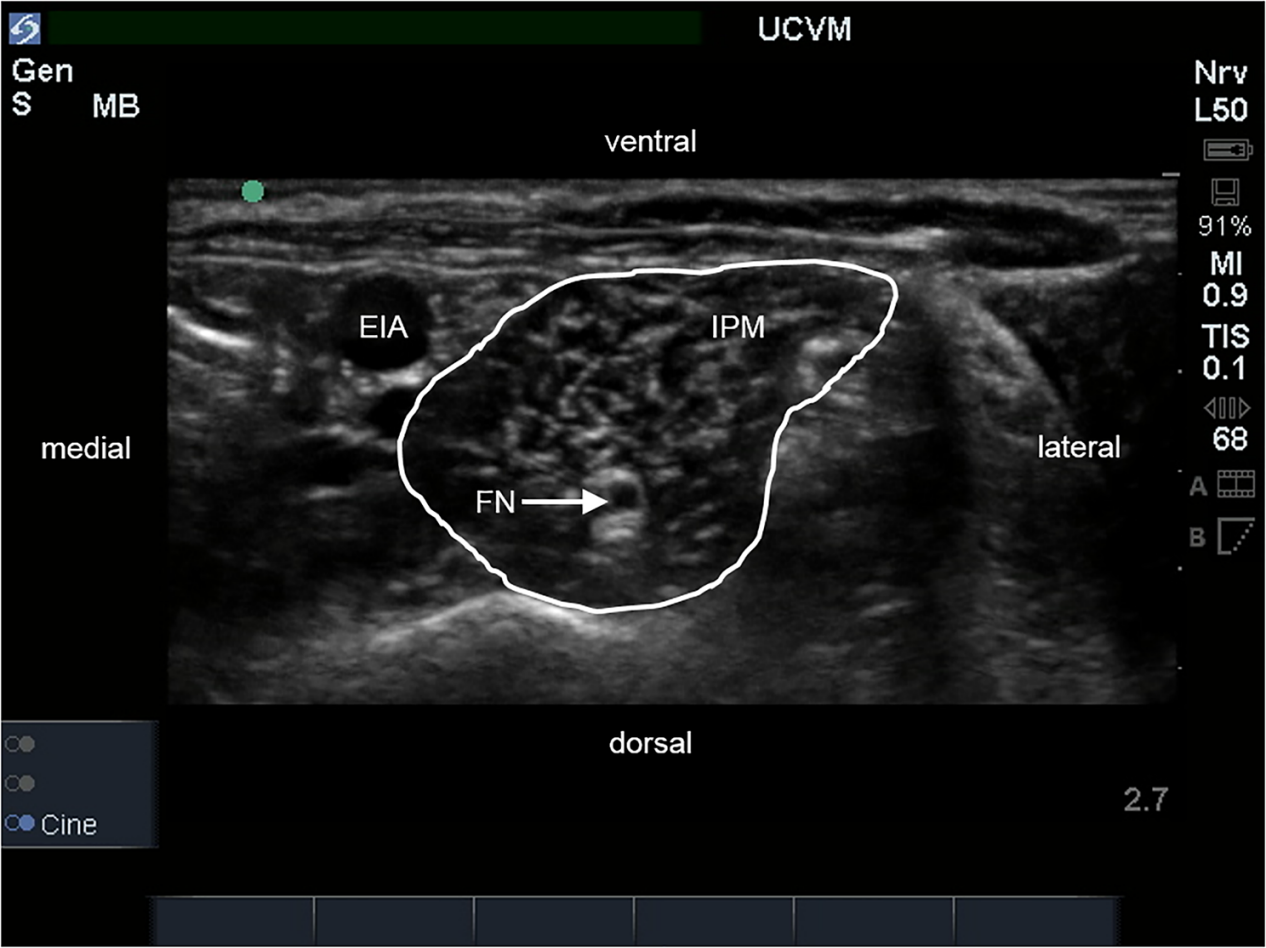
Ultrasound image of the iliopsoas muscle (IPM) in transverse section. The external iliac artery (EIA) is located medial to the IPM and the femoral nerve (FN) is identified as a round hypoechoic structure within the muscle.

**Fig 2:**
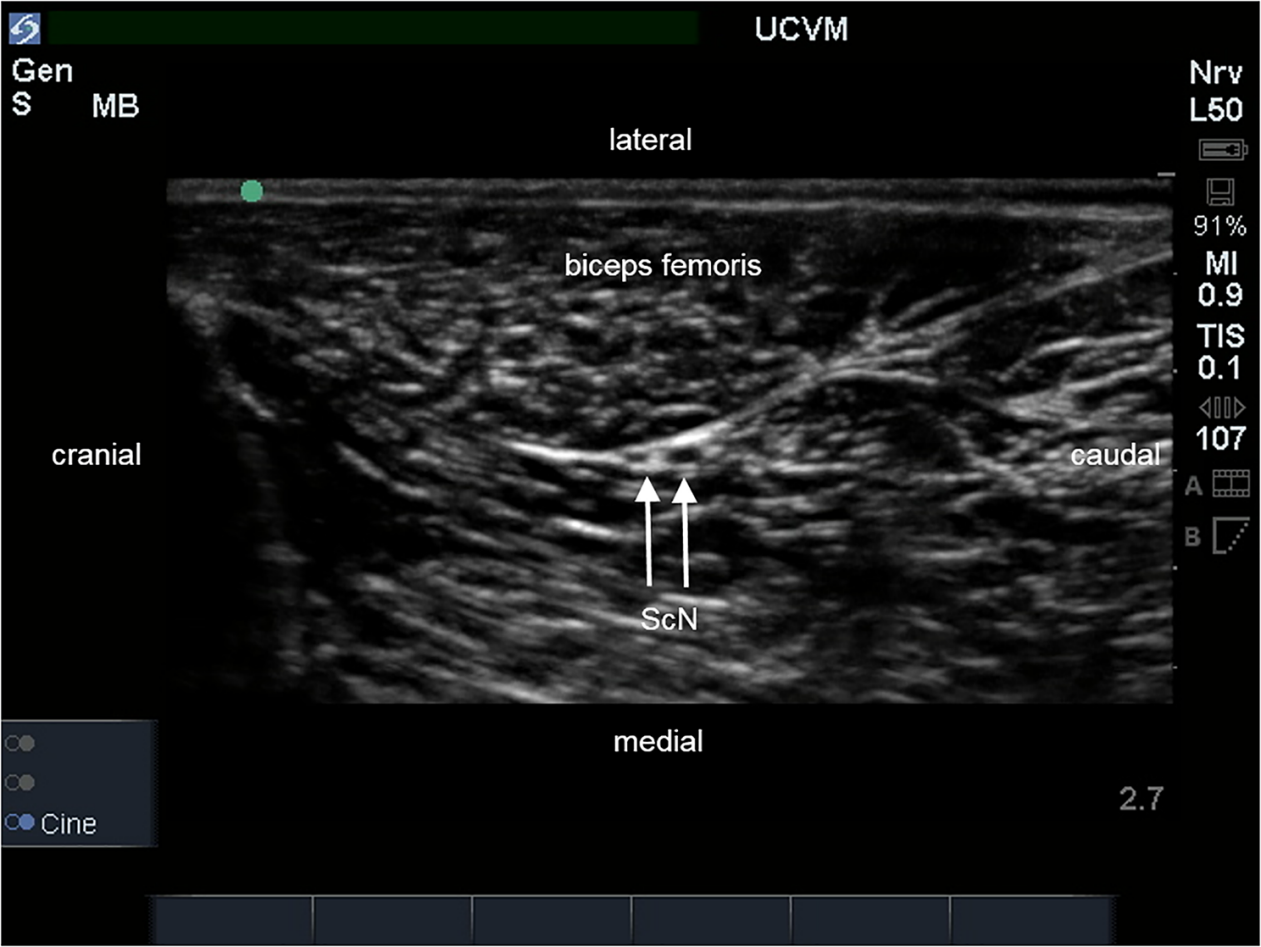
Ultrasound image of the lateral aspect of the pelvic limb. The sciatic nerve (ScN) is located medial to the biceps femoris muscle and is identified as two ovoid hypoechoic structures with the cranial component (peroneal nerve) being smaller than the caudal component (tibial nerve) in transverse section.

The ultrasound-guided approaches that were used in this study allowed for identification of the FN, ScN, observation of needle movement, and the spread of injectate around the target nerves in all cases. The iliopsoas muscle appeared as previously described as an oval, hypoechoic structure with an internal pattern of scattering echoes. The FN appeared as a single, rounded hypoechoic structure with a thin hyperechoic rim within the body of the iliopsoas muscle (Fig 1). Electrostimulation of the FN at a minimum threshold stimulating current between 0.4 and 0.5 mA consistently produced contraction of the quadriceps femoris muscle and extension of the stifle when the tip of the needle was within close proximity of the nerve. The ScN appeared as two, rounded hypoechoic structures with thin hyperechoic rims between the muscles of the thigh where it lies medial to the biceps femoris muscle and caudal to the femur (Fig 2). The two components of the ScN were readily distinguished and the cranial structure, the common peroneal nerve, always appeared smaller in diameter than the caudal structure, the tibial nerve. Electrostimulation of the two components of the ScN at a minimum threshold stimulating current between 0.4 and 0.5 mA consistently produced extension (stimulation of the tibial nerve) or flexion (stimulation of the common peroneal nerve) of the tarsus, respectively, when the tip of the needle was within close proximity to the nerves.

The spread of bupivacaine was visualized surrounding the target nerves in all cases. Differences in distribution were observed between the two blocks: when performing the FN block, we were able to achieve a circumferential spread of the bupivacaine around the nerve, while in the case of the ScN, the distribution of the injectate did not encompass the whole nerve and spread of bupivacaine was consistently less on the lateral side of the ScN where it is in close contact with the fascia of the biceps femoris muscle.

There were no differences in the mean plasma concentrations of bupivacaine that resulted from the two blocks at any time point over the first 60 minutes (Table 1 and Table 2), however, the plasma concentration of bupivacaine at five minutes was significantly higher than the concentration measured at 60 minutes for each block (FN block *P* = 0.0011; ScN block *P* = 0.0012). No dog had a bupivacaine concentration greater than 0.78 μg mL^-1^ at any time point.

**Table 1:** Plasma bupivacaine concentrations (μg mL^-1^) following femoral nerve blocks.

Time after injection (min)	Dog 1	Dog 2	Dog 3	Dog 4	Dog 5	Dog 6
**5**	0.40	0.13	0.25	0.14	0.23	0.25
**10**	0.36	0.17	0.25	0.11	0.14	0.15
**15**	0.32	0.13	0.13	0.06	0.10	0.14
**20**	0.28	0.17	0.30	0.06	0.12	0.14
**30**	0.26	0.17	0.26	0.06	0.09	0.11
**60**	0.15	0.07	0.20	0.06	0.05	0.10

**Table 2:** Plasma bupivacaine concentrations (μg mL^-1^) following sciatic nerve blocks.

Time after injection (min)	Dog 1	Dog 2	Dog 3	Dog 4	Dog 5	Dog 6
**5**	0.06	0.11	0.78	0.03	missing	missing
**10**	0.06	0.12	0.55	0.03	0.03	0.05
**15**	0.11	0.12	0.44	0.06	0.18	0.06
**20**	0.16	0.12	0.44	0.05	0.03	0.06
**30**	0.14	0.10	0.39	0.04	0.04	0.07
**60**	0.14	0.13	0.29	0.02	0.03	0.07

All dogs developed complete loss of sensory and motor function following perineural injection of bupivacaine around the FN and ScN. Dogs showed motor and proprioceptive deficits, including ataxia and the inability to bear weight and use the blocked limbs and, in the areas innervated by the SaN and ScN, all injections resulted in complete sensory blockade of the respective dermatomes.

Inter-rater agreement was high after the blinded assessors were trained to use the rating instrument on nine sample videos. Compared to the ‘gold standard’ scores that were assigned by the primary investigators who assessed the dogs at each time point in person, the weighted kappa values (95% CI) for the degree of motor and sensory block for each of the blinded raters were: Rater 1, 0.73 (0.45–1.0) and 0.83 (0.61–1.0); and Rater 2, 0.83 (0.61–1.0) and 0.86 (0.66–1.0). Based on the high level of inter-rater agreement that was achieved, no further training was conducted and the blinded assessors were provided with the full catalogue of video recordings to evaluate. Moderate to very good agreement was observed between the blinded assessors when they evaluated the 144 video recordings that documented the degree of sensory and motor deficits following the two nerve blocks. The weighted kappa values (95% CI) for evaluating the degree of motor and sensory function were 0.86 (0.78–0.95) and 0.52 (0.38–0.67) for the FN blocks, and 0.87 (0.80–0.94) and 0.52 (0.36–0.67) for the ScN blocks, respectively.

The median (range) times to complete recovery from motor deficits for the femoral and sciatic nerve blocks were 11 (6–14) hours and 12 (4–18) hours, respectively. The median (range) times to complete recovery from sensory deficits for the femoral and sciatic nerve blocks were 15 (10–18) hours and 10 (4–12) hours, respectively. Based on the scoring of limb dysfunction, our hypothesis that FN blocks would be of shorter duration than ScN block was rejected as there were no differences in the duration of motor or sensory blockade following the two nerve blocks. All dogs had complete recovery of both motor and sensory function at a maximum of 18 hours post injection.

## Discussion

Surgical procedures of the pelvic limb are common in small animal practice. The ScN is responsible for most of the sensory innervation to the lateral, caudal, and part of the cranial aspects of the limb and, after the FN arises from the iliopsoas muscle, the SaN branches from the FN and is responsible for the sensory innervation of the medial aspect of the limb [11]. Since the FN and the ScN are the primary nerve supplies to the pelvic limb in the dog, various methods for blocking these nerves with local anesthetics have been systematically developed in attempt to improve perioperative analgesia for patients undergoing painful surgical procedures involving the pelvic limb. Previous reports have described combining an injection around the ScN with either a proximally-located injection of the lumbar plexus, or a more distally-located injection around either the FN or the SaN in the inguinal region [5–8,12,13]. Regardless of the particular technique that is used, the nerve blocks can be used as either the principal anesthetic or, more commonly, as adjuncts to general anesthesia [1–4,9,17,20,21]. If a local anesthetic of sufficient duration is selected, nerve conduction blockade has the potential to provide substantial post-operative pain relief as well [1–3,17].

The success of any sensory nerve block depends on placement of the local anesthetic in close proximity to the nerve that serves the target area. Compared with blind techniques, the use of electrostimulation and/or ultrasound guidance offers the advantages of being able to confirm the identity of the nerve using physiological or visual cues, respectively, prior to drug administration. The combined electrostimulation and ultrasound-guided caudal approach to the ScN that was used in this study is well described and is commonly employed [5–8]. Using this technique, the ScN was easy to identify at the level of the mid-femur in its location medial to the thickest part of the biceps femoris muscle, caudal to the femur and cranial to the semimembranosus muscle. On short-axis view, it appeared on ultrasound as two round, hypoechoic structures surrounded by hyperechoic connective tissue and we were able to visualize the advancement of the needle towards the nerve, confirm the identity of the nerve using electrostimulation, and observe the real time spread of local anesthetic as it was being injected. Ultrasound also allowed us to avoid making intravascular or intraneural injections, and, consistent with previous reports, we achieved 100% success in inducing complete sensory and motor blockade of the ScN in each dog using this approach.

Studies that have described blocking the FN in dogs at the level of the femoral triangle report variable success rates even when nerve stimulation and/or ultrasound visualization were employed [5–7,20]. The variable success rates of this approach may be due to the local anesthetic solution being deposited on the wrong side of the fascia iliaca. Since electrostimulation is able to elicit a motor response through the fascial plane, if the needle tip does not actually puncture the fascia, the local anesthetic will be deposited in the wrong location and the nerve block will fail. Despite this technical uncertainty, even if the FN is accurately identified and blocked at this location, the SaN and branches from the other nerves that potentially provide sensory input to the pelvic limb (e.g. ON, LCFN) may be missed, resulting in a partial or failed block depending on the nature of the surgical procedure and a patient’s specific innervation. Furthermore, due to the close proximity of the FN to the femoral artery and vein in the inguinal region, inadvertent vascular puncture is a risk when a needle is placed into the femoral triangle. Given these considerations, there has been increasing interest in developing techniques to block the FN at the level of the lumbar plexus, either as a single injection into the iliopsoas muscle, or as multiple injections that target the individual nerve roots of L4, L5 and L6 [10,12,13,15–17]. Compared with the originally-described approaches to blocking the FN at the femoral triangle, blocking the FN within the iliopsoas muscle may also be technically easier to perform and there is no risk of inadvertent vascular puncture [12,14,16,17]. Using the ultrasound-guided approaches described by Mahler and Echeverry, we were able to visualize the FN, the advancement of the needle, and the spread of local anesthetic around the nerve [10–12]. Since the FN can appear similar to muscle fascicles within the iliopsoas muscle, we used electrostimulation to confirm the identity of the FN before injection of the local anesthetic. Using these techniques, we achieved 100% success in inducing complete sensory and motor block of the FN in each dog.

Bupivacaine is one of the most commonly used local anesthetics for regional anesthesia in small animals since it is readily available in many jurisdictions and is relatively safe at recommended doses. Consistent with previous reports, the spread of bupivacaine was visualized using ultrasound in all cases as an anechoic space surrounding the nerve. While circumferential spread of the local anesthetic was documented during performance of the FN blocks, as has been previous described, with the ScN blocks the distribution did not encompass the entire nerve [8].

Previous studies on the use of FN and ScN blocks in small animals have used lidocaine, ropivacaine, or bupivacaine, but the expected durations of sensory block (for pain) and motor block (for muscle relaxation) that are induced by bupivacaine are not widely reported [1,4–9,16,21,22]. The results of this study show that use of bupivacaine for FN and ScN blocks using the techniques described will induce comparable levels of clinically-relevant sensory and motor blockade in the affected limb for up to 20 hours following injection. Our observation that there were no differences in the duration of motor and sensory dysfunction between the two blocks is consistent with previous laboratory studies that used bupivacaine or ropivacaine for FN and ScN blocks in dogs [7,16,22]. Based on the expected durations of most pelvic limb surgical procedures, as performed in this study, these peripheral nerve blocks would be expected to contribute to balanced anesthesia for the surgical procedure as well as analgesia into the postoperative period. These results are similar to those of previous studies that reported on the duration of analgesia following FN and ScN blocks in actual patients that found clinically-relevant analgesic effects and proprioceptive deficits for approximately 10 hours [2,4,9,16,17].

Our hypothesis that bupivacaine levels would be higher following FN block was rejected. Our study tested a volume of 0.15 mL kg^-1^ for each block, which is equivalent to a dose of 0.75 mg kg^-1^ with the 0.5% bupivacaine solution that was used. At that dose, there were no differences at any time between the plasma concentrations of bupivacaine that resulted from administration at either an intramuscular or an interfascial site, and no signs of adverse cardiovascular effects were detected while the dogs were being monitored over 60 minutes following drug administration. The highest plasma bupivacaine concentration that was measured in any dog was 0.78 μg mL^-1^, which is well below the level that is associated with signs of systemic toxicity in either awake or anesthetized dogs. Liu et al. (1982) found that bolus intravenous administration of bupivacaine up to 1 mg kg^-1^ in pentobarbital anesthetized dogs resulted in no changes in heart rate, mean arterial blood pressure, stroke volume, or cardiac output [23]. Subsequent studies have shown that intravenous administration of bupivacaine at ~ 4–5 mg kg^-1^ would induce seizures in awake dogs and that two-times the convulsive dose would cause respiratory arrest and cardiovascular collapse resulting in death [24,25]. In awake dogs, onset and recovery from seizures are associated with plasma bupivacaine concentrations ~18.0 μg mL^-1^ and ~3.22 μg mL^-1^, respectively and onset of cardiovascular collapse occurs at plasma bupivacaine concentrations ~5.7 μg mL^-1^ [25,26]. Based on those data and the techniques that were used to perform the nerve blocks in our study, using 0.15 mL kg^-1^ of bupivacaine would not be expected to be associated with any increases in patient risk. However, as is recommended with any regional anesthetic technique, the patient should be monitored closely following administration of the local anesthetic in order to detect and appropriately treat any abnormal clinical signs. Additional studies of local anesthetic uptake will be required when other new peripheral nerve blocks are developed, as well as to further explore how the use of ultrasound might allow for even lower doses of local anesthetic to be used without affecting the overall efficacy of these blocks.

There are several limitations to this study. The nerve blocks were performed on a relatively small sample size of healthy dogs that did not undergo surgical procedures. Since the dogs were anesthetized, we were unable to assess the onset time for the blocks. This was not a primary goal of our study and the scenario we used is similar to clinical practice whereby a patient receiving a FN or ScN block for surgery would not normally be expected to recover from anesthesia within an hour. Although our results provide a reasonable picture of what to expect following performance of these nerve blocks in dogs, if they are used in clinical practice, patients should be monitored closely for break-through pain and motor dysfunction on a case-by-case basis. As well, since we wanted to learn about the potential for systemic toxicity and did not want to introduce confounding factors into the uptake of bupivacaine from the injection sites, only isoflurane was used to anesthetize the dogs. Further studies about local anesthetic absorption when more than one agent is used for balanced anesthesia are required.

Based on the more obvious physical signs that were used to evaluate motor function, assessment of motor blockade appeared to be easier to score, as was reflected in the higher levels of inter-rater agreement for motor function compared to sensory function. Since the dogs that were used in the study are primarily used for teaching basic handling skills and procedures to veterinary students, they are very calm and stoic. At some time points, certain dogs showed very little response to skin pinching while their sensory function was being tested. Even the primary investigators who were performing the skin pinching found it difficult to assess the dogs’ responses to stimulation at these times. Differences in patient demeanor likely contributed to the lower levels of inter-rater agreement for sensory function when the two types of video recordings were evaluated. In hindsight, testing the responses to skin pinching at another location would have allowed for a comparison of responses and a potentially better assessment of sensory blockade. For postoperative analgesia to be evaluated more objectively, future studies should consider using another method to assess sensory function if their research subjects are similarly stoic. Regardless, we were able to document obvious clinical effects in our dogs that would last into the typical postoperative period and our results are consistent with previous reports that describe sensory blockade for approximately 12 hours following bupivacaine administration.

While we achieved 100% success with our blocks, as with any technical skill, there is a learning curve and other operators might not initially achieve the same level of success with their blocks. However, the approaches that were used in this study are easy to master and since they are not associated with much anatomical risk, they are good techniques to consider using for any pelvic limb procedure distal to the mid-femur.

The results of this study suggest that when a local anesthetic is injected around the FN and ScN using the techniques described, successful motor and sensory nerve blocks can result. The described approaches are feasible, allow for the visualization of the target nerves at locations away from important vascular structures, and did not result in complications. At the doses of bupivacaine that were used, these blocks can last approximately 12 hours, patients are fully recovered by 18 hours, and the blocks do not appear to be associated with any risk of systemic toxicity as a result of uptake of the local anesthetic from the sites of injection. Further research is required in order to learn how these techniques might benefit clinical patients that are undergoing painful surgical procedures.

## Supporting information

S1 Table Blood concentrations of bupivacaine following femoral and sciatic nerve blocks in dogs. (XLSX)

S2 Table Duration of clinical effects of femoral and sciatic nerve blocks in dogs. (XLSX)
